# Application and mechanism study of EMD-Gel composite scaffold in dental pulp tissue repair

**DOI:** 10.3389/fbioe.2025.1739495

**Published:** 2025-12-10

**Authors:** Luchen Gui, Peimeng Zhan, Qian Zeng, Zhuoyao Liang, Jiaxin Zou, Jiang Guo, Jiacheng Lin

**Affiliations:** 1 Hospital of Stomatology, Guanghua School of Stomatology, Sun Yat-Sen University, Guangzhou, China; 2 Guangdong Provincial Key Laboratory of Stomatology, Guangzhou, China

**Keywords:** enamel matrix derivatives, gelatin methacryloyl, reparative dentinogenesis, anti-inflammatory and odontogenic synergy, pulp repair

## Abstract

This study developed an enamel matrix derivative-gelatin methacrylate (EMD-Gel) composite scaffold for functional dental pulp regeneration, addressing the limitations of traditional capping materials in inflammation control. The photo-crosslinked EMD-Gel exhibited a porous structure and sustained amelogenin release. *In vitro*, it promoted human dental pulp stem cell (hDPSCs) differentiation and inhibited inflammation. *In vivo* (rat pulp capping), EMD-Gel generated a continuous reparative dentin bridge of 213.3 ± 9.5 µm and exhibited substantially lower inflammatory infiltration than iRoot BP Plus, highlighting its advantages in both dentin bridge quality and inflammation resolution. Mechanistically, EMD-Gel synergistically activates the Wnt/β-catenin pathway and inhibits *CCL2*-*MMP3*-mediated inflammation. This material provides a novel, dual-functional platform for clinical pulp therapy.

## 1 Introduction

Pulpitis is a common infectious dental disease that impairs masticatory function and may contribute to systemic complications such as endocarditis, posing a significant public health burden (Khijmatgar et al., 2024). Pulp capping is a crucial therapeutic modality for vital pulp preservation (Duncan, 2022). The procedure aims to cover exposed or caries-adjacent pulp with bioactive materials, effectively eliminating infection, isolating irritants, and inducing the underlying pulp to generate a reparative dentin barrier (Islam et al., 2023). This procedure applies bioactive materials to exposed or caries-adjacent pulp to eliminate infection, isolate irritants, and stimulate the formation of a reparative dentin barrier (AAE Position Statement on Vital, 2021).

Human dental pulp stem cells (hDPSCs) are the key progenitor cells mediating the regeneration of the dentin-pulp complex (Sultan et al., 2025), and their differentiation capacity dictates the efficacy of regenerative therapy. Consequently, regulating hDPSC differentiation, regeneration, and immunomodulatory capacity has become a central focus in recent regenerative research (Koh et al., 2021). A key strategy for functional dentin–pulp regeneration is to integrate the essential characteristics of ideal capping materials, such as immunomodulation, enhanced repair capacity, and clinical manageability (Nie et al., 2021; Dal-Fabbro et al., 2023). This necessity has made constructing a microenvironment that is both biologically inductive and structurally stable a key area in regenerative dentistry (Hu et al., 2023).

Although modern capping materials such as bioceramics (e.g., iRoot BP Plus and MTA) can achieve clinical success rates above 80%, they still present fundamental limitations (AAE Position Statement on Vital, 2021). Their high rigidity and lack of porosity after setting critically impede the migration and organized regeneration of DPSCs (Rajasekar et al., 2025). Furthermore, the resulting dentin bridge often exhibits structural anomalies or inadequate interfacial sealing, posing a long-term threat of microleakage (Prasad Kumara et al., 2025). Therefore, the development of new materials is not aimed at simple substitution, but at overcoming these persistent clinical limitations to ensure more predictable, higher-quality regenerative outcomes in complex scenarios.

Enamel matrix derivative (EMD, Emdogain®), which is rich in amelogenin and other bioactive components (Quiroga et al., 2025), has been investigated for pulp capping due to its potent bioactivity in promoting cell differentiation and tissue regeneration during tooth development (Tsuchida and Nakayama, 2023). Ample evidence confirms that EMD effectively promotes the odontogenic differentiation of DPSCs and modulates inflammation (Sordi et al., 2022; Panahipour et al., 2022; Zhang et al., 2022a). However, its direct application is problematic due to the washout and unstable concentration associated with its liquid formulation (Fraser et al., 2022; Miron et al., 2016). Additionally, its high cost has not consistently demonstrated clear biological superiority over existing bioceramics. The major obstacle limiting the clinical translation of EMD is the absence of a delivery system that can maintain an effective local concentration within the pulp cavity and guide organized tissue regeneration (Huang et al., 2024). Although previous studies have attempted to combine EMD with various carriers such as bioceramics (Al‐Hezaimi et al., 2013), PRF (Aydemir Turkal et al., 2016), or statin-modified matrices (Karanxha et al., 2013), these systems are generally limited by the intrinsic rigidity of the materials, insufficient microstructural adaptability, or the inability to maintain a stable local concentration of bioactive proteins within the pulp cavity (Duarte et al., 2025). To date, only limited efforts have addressed the development of a biomimetic, photocrosslinkable, degradable hydrogel platform capable of achieving spatiotemporal controlled release of EMD for pulp regeneration. This work therefore fills a critical translational gap by introducing an EMD-loaded GelMA hydrogel that not only stabilizes EMD *in situ* but also reconstructs a biologically permissive microenvironment capable of simultaneously modulating inflammation and promoting odontogenic differentiation. These functional advantages cannot be achieved by EMD or GelMA alone.

Based on these challenges, this study proposes constructing an EMD-gelatin methacrylate (GelMA) composite scaffold system. The central rationale is to load EMD onto a biomimetic hydrogel scaffold (Kim et al., 2012) to overcome its limitations as a standalone liquid preparation. GelMA provides a stable microenvironment (Karanxha et al., 2013), which facilitates DPSC adhesion and proliferation while enabling the controlled and sustained release of EMD. We hypothesize that this composite will not only resolve EMD’s application issues but, through the synergy of bioactive signals and the physical scaffold (Yuan et al., 2023), will exhibit superior regenerative potential by inducing a more structurally complete reparative dentin compared to mineralization reliant solely on a physical barrier.

To validate this hypothesis, this study first systematically assessed the physicochemical properties of the EMD-Gel scaffold. We established iRoot BP Plus as the control in both *in vitro* and *in vivo* experiments—a comparison crucial for objectively determining the new material’s relative benefits. Using a rat pulp capping model, we monitored the short-term (4-day) inflammatory response and evaluated the long-term (28-day) quality of dentin bridge formation to comprehensively compare the material’s impact on healing against the existing standard. Finally, RNA sequencing (RNA-seq) was employed to deeply investigate the molecular mechanisms by which the scaffold regulates cellular behavior. We anticipate this collective work will provide the theoretical and experimental foundation for developing novel pulp capping strategies based on active factor delivery.

## 2 Results

### 2.1 Preparation, main components, and related mechanisms of EMD-Gel materials

**FIGURE 1:**
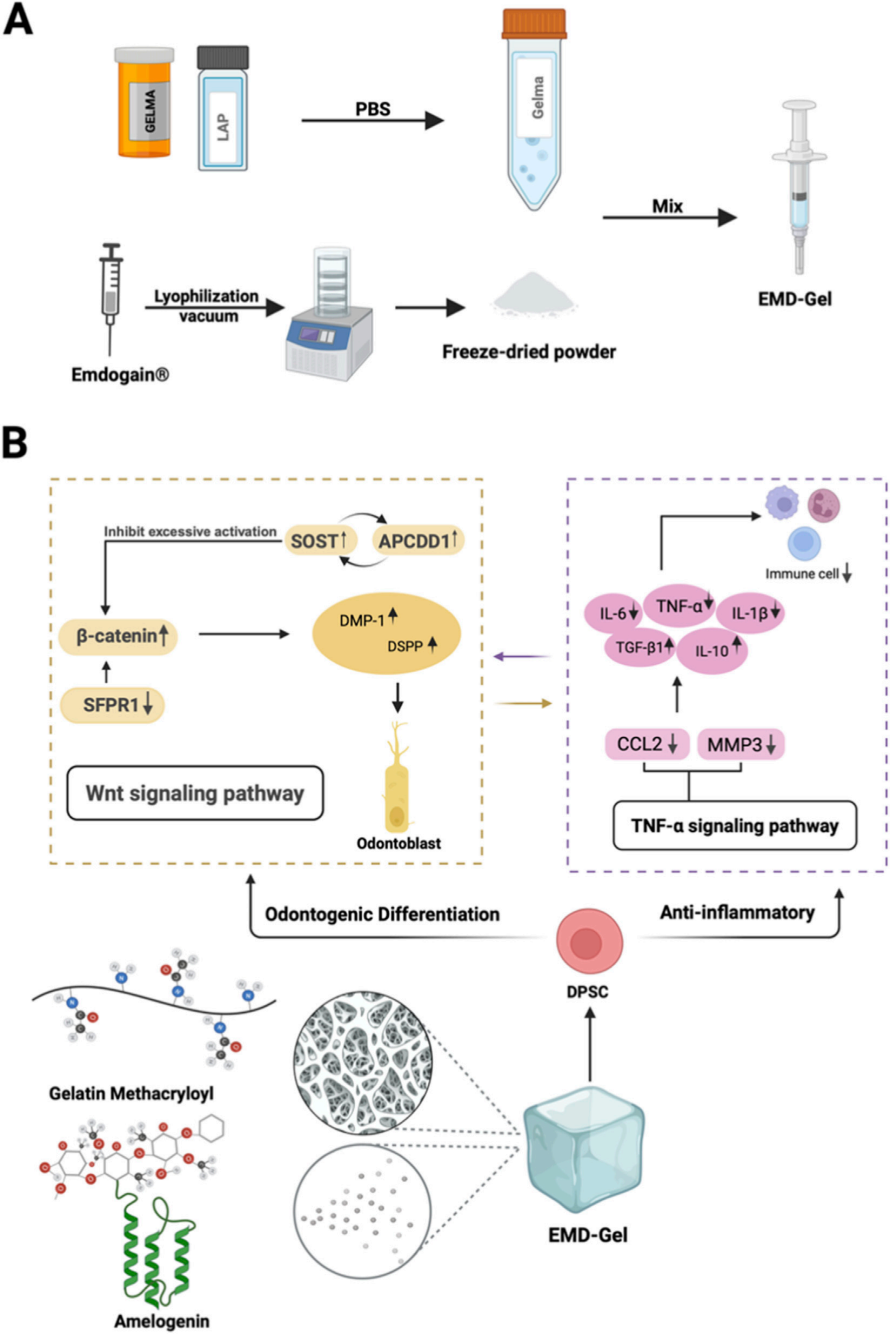
**(A)** Visually illustrates the preparation route of EMD-Gel; **(B)** EMD-Gel composed of GelMA and amelogenin regulates DPSC differentiation and inflammation through Wnt/TNF pathway synergy.

Figure 1A illustrates the preparation workflow of the EMD-Gel composite, highlighting its suitability for applications in pulp repair therapies. The material is primarily composed of gelatin methacryloyl (GelMA) and Amelogenin. As shown by RNA-sequencing analyses, these components act synergistically to enhance the odontoblastic differentiation of dental pulp stem cells (DPSCs) while concurrently exerting anti-inflammatory effects through modulation of the Wnt and TNF signaling pathways. Together, these actions help establish a microenvironment that is highly supportive of pulp-tissue regeneration (Figure 1B).

### 2.2 Characterization of the EMD-Gel material

**FIGURE 2:**
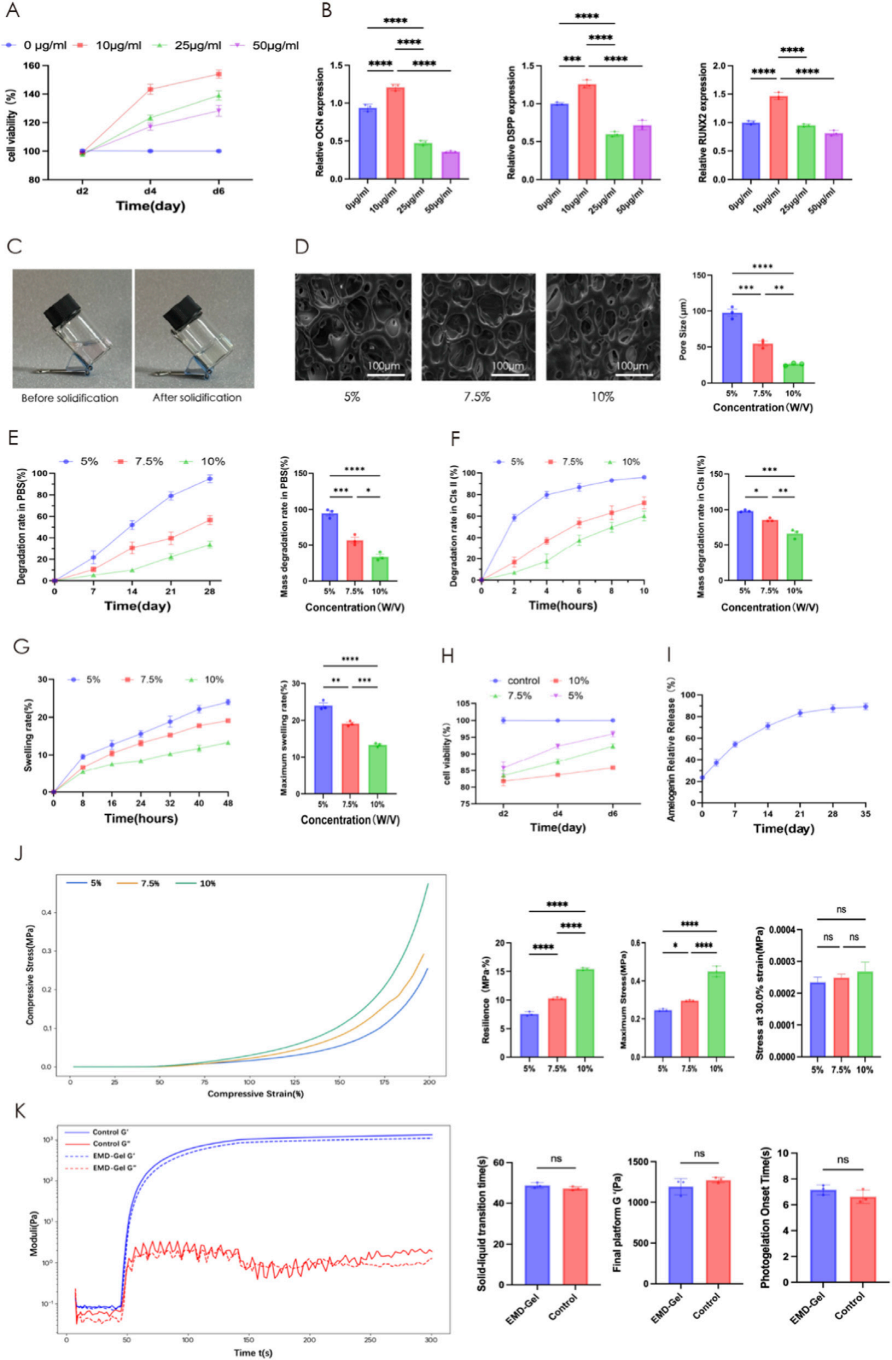
**(A)** Effect of EMD concentration on DPSC proliferation; **(B)** qRT-PCR analysis of dentinogenic gene expression in hDPSCs treated with different concentrations of EMD; **(C)** Photographs of EMD-Gel before and after gelation; **(D)** SEM images of the porous microstructure of EMD-Gel; **(E)** Degradation profile and cumulative degradation rate in PBS; **(F)** Enzymatic degradation in type II collagenase; **(G)** Swelling curve and maximum swelling ratio; **(H)** DPSC proliferation in EMD-Gel hydrogels with varying crosslinking densities; **(I)** Cumulative amelogenin release profile; **(J)** Mechanical properties of EMD-Gels with different crosslinking densities; **(K)** Rheological analysis of photocrosslinking behavior. (*p < 0.05, **p < 0.01, ***p < 0.001; scale bar = 100 μm).

An EMD concentration of 10 μg/mL resulted in a significantly higher relative cell proliferation rate (p < 0.01) and a significant increase (p < 0.001) in odontoblastic differentiation gene expression, including *OCN, DSPP, RUNX2* and other key markers, indicating that 10 μg/mL represents the optimal biological concentration for promoting hDPSC activity (Figures 2A,B). The EMD-Gel precursor remained completely fluid at 37 °C and rapidly converted into a mechanically stable hydrogel upon photocrosslinking, demonstrating favorable operability for clinical manipulation (Figure 2C). SEM visualization showed an interconnected honeycomb-like porous microstructure, with the 5% EMD-Gel exhibiting the most suitable pore size (86.94 ± 11.30 μm), providing channels that effectively support nutrient transport and metabolic waste exchange without imposing diffusion barriers to DPSC survival, a microstructural advantage consistent with the enhanced cell viability observed in the CCK-8 assay (Figure 2D). The 5% EMD-Gel degraded almost completely within 4 weeks in PBS (94.9% ± 6.26%), suggesting minimal risk of long-term material retention and demonstrating a degradation profile compatible with the overall timeline of pulp-tissue regeneration (Figure 2E). Enzymatic degradation in type II collagenase further demonstrated rapid responsiveness, with the 5% EMD-Gel reaching 98.4% ± 1.4% degradation within 10 h, suggesting an inflammation-responsive degradation behavior that supports microenvironmental remodeling during the early stages of pulp healing (Figure 2F). Swelling analysis showed that the 5% EMD-Gel exhibited the highest equilibrium swelling ratio (24.1% ± 1.23%), significantly surpassing the 7.5% (19.12% ± 0.7%, p < 0.01) and 10% (13.3% ± 0.5%, p < 0.001) groups, indicating improved interfacial adaptation for achieving stable coronal sealing (Figure 2G). CCK-8 results demonstrated that DPSC proliferation was most robust in the 5% EMD-Gel group (91.4% ± 5.2%), significantly exceeding that in the 7.5% and 10% groups (p < 0.001), supporting its superior cytocompatibility (Figure 2H). ELISA quantification revealed a sustained and controlled biphasic release profile of amelogenin, with cumulative release reaching 87.7% ± 3.5% by day 28 and stabilizing around the biologically effective level of approximately 10 μg/mL (Figure 2I). Mechanical characterization showed comparable elastic modulus among gel concentrations under 30% strain, while the 5% EMD-Gel displayed adequate maximum stress resistance (0.2540 ± 0.006 MPa) and toughness (7.9914 ± 1.352 MPa·%), indicating that the 5% formulation can tolerate the moderate compressive forces typically applied during pulp-capping manipulation and early clinical loading (Figure 2J). Rheological assessment confirmed that G′ rapidly surpassed G″ during light exposure, demonstrating fast gelation kinetics, and the incorporation of EMD did not alter the photocrosslinking behavior, with the final G′ plateauing around 10^3^ Pa (Figure 2K).

### 2.3 Anti-inflammatory and odontogenic effects of EMD-Gel on LPS-stimulated hDPSCs

**FIGURE 3:**
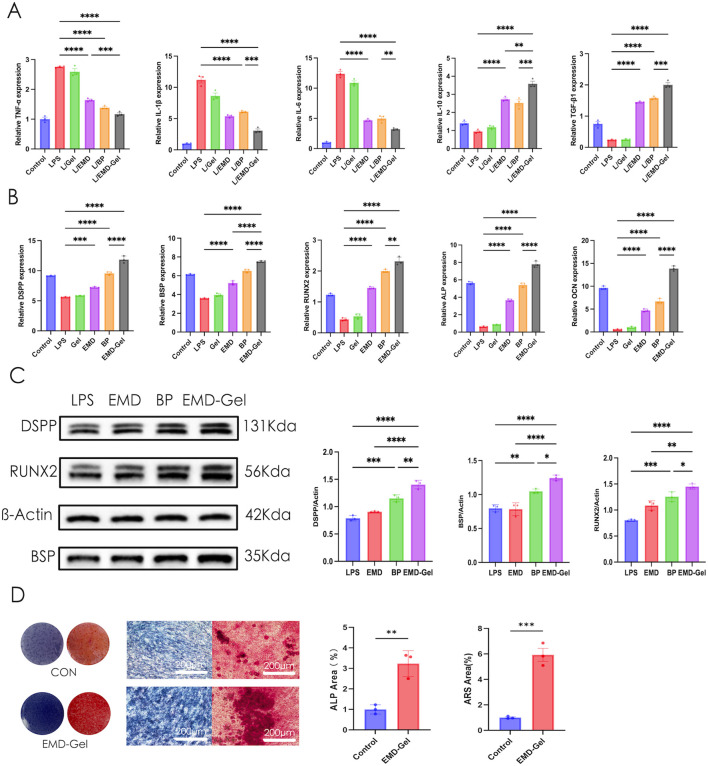
**(A)** Effect of LPS on inflammatory gene expression in different groups detected by qRT-PCR; **(B)** Odontogenic gene expression of LPS-challenged hDPSCs detected by qRT-PCR; **(C)** Expression of DSPP, BSP, and Runx2 proteins detected by Western blot; **(D)** ALP and ARS staining comparing the mineralization capacity of hDPSCs in the presence or absence of EMD-Gel. (*p < 0.05, **p < 0.01, ***p < 0.001).

In an LPS-induced inflammatory model using hDPSCs, EMD-Gel treatment resulted in a 2.3- to 3.8-fold reduction in pro-inflammatory cytokines (*IL-1β, TNF-α, IL-6*) compared with the LPS group (p < 0.01), while simultaneously producing significantly higher levels of anti-inflammatory mediators (*TGF-β1, IL-10*) than the EMD-alone group (p < 0.05) and exhibiting a stronger anti-inflammatory response than the BP control (p < 0.01). No significant differences were observed in the GelMA group relative to the LPS group (p > 0.05), confirming that the bioactivity was dependent on the incorporation of EMD (Figure 3A). In the same LPS-induced inflammatory model, odontogenic gene expression analysis showed that the EMD-Gel group exhibited significantly higher transcription of key differentiation markers than both the EMD-alone and BP groups (p < 0.01), whereas EMD alone induced only moderate upregulation and GelMA showed no independent activity (Figure 3B). Western blot analysis showed that EMD-Gel markedly increased DSPP, BSP, and Runx2 protein expression compared with the LPS group, yielding a 1.3- to 2.1-fold elevation (p < 0.01). In addition, the protein levels in the EMD-Gel group were generally comparable to or slightly higher than those observed in the BP group, together indicating effective restoration of odontogenic protein expression under inflammatory conditions (Figure 3C). After 21 days, ALP and ARS staining revealed significantly enhanced mineralization in the EMD-Gel group, with a 3.2-fold larger ALP-positive area (p < 0.01) and a 5.9-fold greater ARS-stained nodule area (p < 0.001) compared with the Control group, demonstrating enhanced matrix maturation and mineral deposition (Figure 3D).

All Western blot bands were obtained from the same membrane, and cropped images with red dividing lines are shown for clarity; densitometric quantification was performed using three independent biological replicates.

### 2.4 Application of the EMD-Gel in a pulp capping model using rat first molars

**FIGURE 4:**
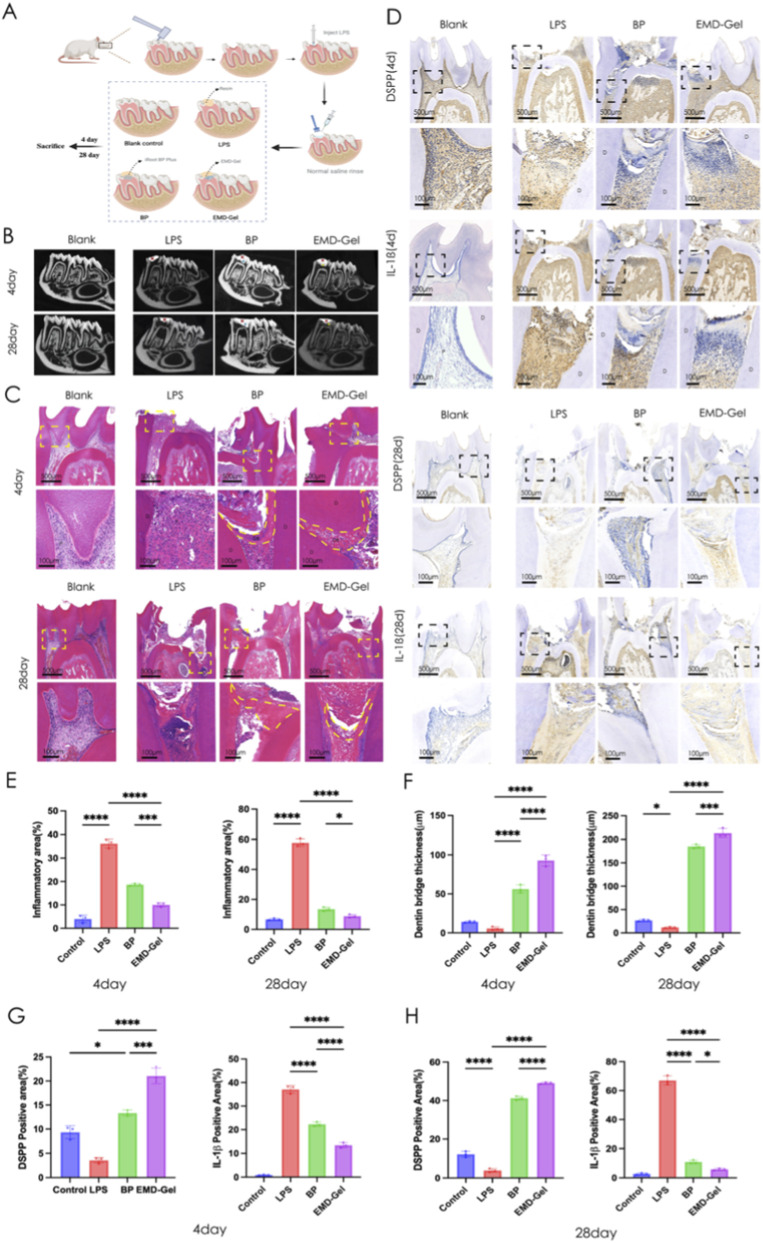
**(A)** Schematic diagram and establishment of the rat pulp-capping model; **(B)** Micro-CT evaluation of coronal sealing and pulp-capping materials across groups; **(C)** HE staining showing inflammatory response and reparative dentin formation at days 4 and 28; **(D)** IHC detection of odontogenic marker DSPP and inflammatory marker IL-1β at days 4 and 28; **(E)** Quantification of inflammation area (%) at days 4 and 28 based on HE staining; **(F)** Quantification of reparative dentin bridge thickness (µm) at days 4 and 28 based on HE staining; **(G)** Quantification of DSPP-positive area (%) based on IHC staining; **(H)** Quantification of IL-1β-positive area (%) based on IHC staining. (*p < 0.05, **p < 0.01, ***p < 0.001; scale bar = 100 μm/500 μm).

A standardized rat pulp-capping model was successfully established, as illustrated in Figure 4A, which presents the experimental grouping structure consisting of the Blank Control group (non-induced inflammation), the LPS-induced inflammation group, the iRoot BP Plus group (positive control), and the EMD-Gel treatment group, as well as the schematic workflow of model construction. Micro-CT reconstruction images further show the placement of materials within the pulp chamber of the rat mandibular first molars, with flowable resin displayed in red, iRoot BP Plus in blue, and EMD-Gel in yellow. All experimental groups achieved comparable pulp chamber exposure and sealing quality, with standardized access cavity dimensions (2.2 ± 0.7 mm) and a continuous and intact coronal resin sealing layer (0.8 ± 0.1 mm), confirming consistency across operative procedures (Figure 4B). At day 4, HE staining showed that the LPS group exhibited extensive inflammatory infiltration throughout the entire pulp, whereas the EMD-Gel group displayed a more localized inflammatory response confined to the coronal pulp, with the radicular pulp remaining structurally intact and without signs of necrosis. By day 28, both the EMD-Gel and BP groups induced reparative dentin formation (Figure 4C). The EMD-Gel group demonstrated well-restored pulp architecture, characterized by a continuous, dense, and uniformly organized reparative dentin bridge that was tightly integrated with the primary dentin, with a thickness of approximately 213.3 ± 9.5 μm. In contrast, the BP group formed only a diffuse, loosely organized calcified layer with irregular morphology and mild residual inflammatory infiltration (Figures 4E,F).

Inflammatory marker analysis showed significantly lower inflammatory factor expression in both the EMD-Gel and BP groups compared with the LPS group (p < 0.01), with the EMD-Gel group exhibiting more effective inflammatory control at the early stage (p < 0.05) (Figure 4D). At day 4, IHC staining revealed that the EMD-Gel group displayed a localized IL-1β immunoreactivity restricted to the area immediately beneath the exposure site, together with early DSPP-positive signals, whereas the LPS group showed strong IL-1β staining and extensive inflammatory infiltration. By day 28, DSPP expression in the EMD-Gel group became markedly positive and was concentrated beneath the reparative dentin bridge, accompanied by near-complete resolution of inflammatory infiltration; in contrast, the BP group displayed discontinuous or scattered DSPP positivity and persistent mild inflammatory cell infiltration (Figures 4G,H).

### 2.5 RNA-sequencing analysis and qPCR validation of EMD-gel–mediated molecular regulation

**FIGURE 5:**
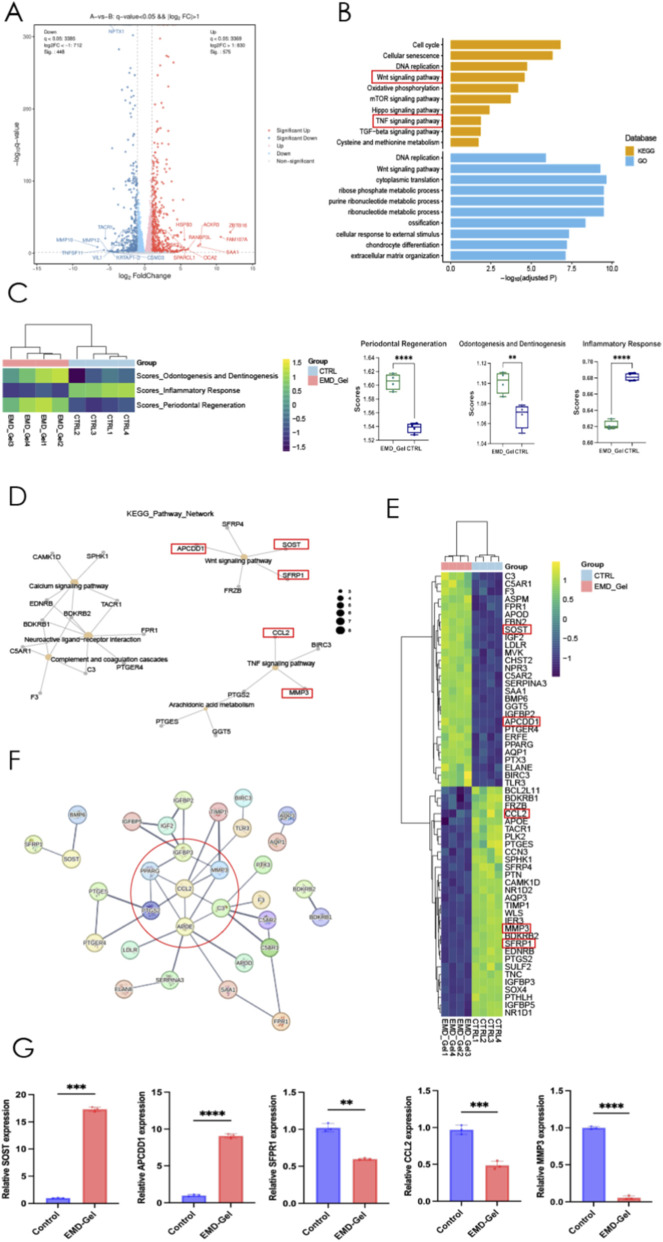
**(A)** Volcano plot of differentially expressed genes between EMD-Gel and Control groups; **(B)** GO and KEGG pathway enrichment map of DEGs; **(C)** Functional score plot showing odontogenic and osteogenic differentiation potential; **(D)** Co-expression network identifying key regulatory modules; **(E)** Cluster analysis of DEGs between EMD-Gel and Control groups; **(F)** Protein–protein interaction (PPI) network diagram; **(G)** RT-qPCR validation of representative DEGs including *SOST, APCDD1, SFRP1, CCL2*, and *MMP3*. (*p < 0.05, **p < 0.01, ***p < 0.001; pathway information adapted from the KEGG database, © Kanehisa Laboratories, with permission).

Volcano plot analysis (|log_2_FC| > 1, p < 0.05) identified a total of 1063 differentially expressed genes (DEGs) between the EMD-Gel and Control groups, including 575 upregulated and 448 downregulated genes, indicating a broad transcriptional response to EMD-Gel treatment and distinct shifts in gene expression profiles (Figure 5A). GO and KEGG pathway enrichment analyses revealed that these DEGs were predominantly enriched in pathways related to Wnt signaling, TNF signaling, and calcium signaling, reflecting transcriptional changes associated with both odontogenic regulation and inflammatory modulation (Figure 5B). Functional scoring further demonstrated that the EMD-Gel group exhibited significantly higher predicted odontogenic and osteogenic differentiation potentials compared with the Control group (Figure 5C). Co-expression network analyses identified several core regulatory gene clusters, including *APCDD1, SOST*, and *SFRP1* within the Wnt pathway, as well as *CCL2* and *MMP3* within the TNF pathway, highlighting coordinated interactions among genes involved in differentiation and inflammation (Figures 5D,E). Protein–protein interaction (PPI) network analysis showed that these key regulators formed interconnected protein modules, with a central hub composed of *SOST, APCDD1, SFRP1, CCL2*, and *MMP3* surrounded by closely interacting partners, indicating that these genes occupy highly connected positions within the regulatory landscape (Figure 5F). qPCR validation further confirmed significantly increased expression of *SOST* and *APCDD1* (p < 0.01) and significantly decreased expression of *CCL2, MMP3*, and *SFRP1* (p < 0.01) in the EMD-Gel group compared with the Control group, closely matching the differential expression trends revealed by transcriptomic sequencing (Figure 5G). Pathway information was obtained from the KEGG database (Kanehisa Laboratories).

## 3 Discussion

This study innovatively developed a methacrylated gelatin hydrogel scaffold loaded with enamel matrix derivative (EMD-Gel) and comprehensively evaluated its anti-inflammatory and odontogenic activities in the context of dental pulp regeneration. Both *in vitro* and *in vivo* experiments confirmed that EMD-Gel effectively regulates the inflammatory microenvironment, induces stem-cell differentiation, and promotes reparative dentin formation. Particularly in a rat pulp capping model (Li et al., 2021), the composite scaffold showed excellent biocompatibility and was able to induce the formation of a continuous and dense reparative dentin bridge, with its quality and thickness being significantly superior to the traditional BP (positive control) group (Yang et al., 2024), thereby demonstrating good potential for clinical translation. The following discussion will delve into three aspects: material design, functional validation, and molecular mechanism.

Since the bioactivity of EMD is significantly affected by its local concentration and stability (Chung and Park, 2017), this study first performed an optimal screening of the effective EMD concentration to prevent cytotoxicity caused by excessively high drug concentration or inefficacy due to low concentration (Stout et al., 2014). The results showed that EMD at 10 μg/mL significantly upregulated the expression of odontogenic differentiation-related genes such as *ALP* and *RUNX2*, whereas higher or lower concentrations led to a weakened biological effect. This finding is consistent with the dose-dependent characteristic reported in previous studies (Qu et al., 2010). After determining the effective concentration of EMD, we further screened the physicochemical ratios of GelMA to obtain a composite scaffold that offers both mechanical adaptability and cytocompatibility (Sakr et al., 2022). We uniformly incorporated 10 μg/mL EMD into GelMA solutions to construct 5%, 7.5%, and 10% EMD-Gel composite systems, which were then systematically evaluated for their morphology, mechanical properties, and biological characteristics.

The results indicated that increasing the GelMA concentration raised the compressive modulus of the scaffolds while significantly reducing their porosity and permeability (Celikkin et al., 2018). The 5% EMD-Gel system possessed a porous structure with an average pore size of approximately 86.94 ± 11.30 μm, which balanced the needs for cell adhesion, oxygen, and nutrient diffusion (Luo et al., 2025). Its elastic modulus (approximately 10^3^ Pa) was sufficient to maintain coronal seal stability (Tapsir et al., 2013), while also exhibiting excellent swelling and photo-crosslinking properties. CCK-8 results further confirmed that the 5% EMD-Gel group was significantly superior to the 7.5% and 10% groups in terms of DPSCs viability (P < 0.01), suggesting that a higher GelMA concentration might restrict cell migration and proliferation due to excessive crosslinking density (Sharifi et al., 2021). Compared to traditional liquid EMD preparations using propylene glycol alginate (PGA) as a vehicle (Jung et al., 2023), the EMD-Gel system not only overcame the drawbacks of insufficient physical strength and easy displacement (De Lauretis et al., 2024) but also enabled rapid curing and precise shaping via photo-crosslinking. Scanning Electron Microscopy (SEM) observation showed that the honeycomb-like porous structure of 5% EMD-Gel provided an appropriate space for DPSC adhesion and migration (Huang et al., 2025). Its degradation period of approximately 4 weeks highly matched the dental pulp repair process, preventing long-term material residue from causing tissue interference (Xiao et al., 2019). Swelling experiments and ELISA results further demonstrated that the release of amelogenin, the main component of EMD (Alp and Ulusoy, 2024), could be sustained in GelMA for 28 days, with a cumulative release rate of approximately 70%. This effectively avoided the loss of activity and local irritation caused by the short-term burst release of EMD (Pramanik et al., 2024; Wang et al., 2018). Based on a comprehensive consideration of mechanical performance, pore structure, and biocompatibility, 5% EMD-Gel was determined as the optimal formulation for subsequent functional studies.

Effective control of inflammation is a crucial prerequisite for successful pulp-capping treatment (Duncan and El‐Karim, 2024). Uncontrolled inflammation can destroy the extracellular matrix (ECM), inhibit the odontogenic differentiation of DPSCs, and even lead to irreversible necrosis (Dai et al., 2024; Shi et al., 2025). In *in vitro* experiments, EMD-Gel exhibited a significant odontogenic induction effect by synergistically modulating inflammation. Specifically, EMD-Gel significantly downregulated the expression of *TNF-α* and *IL-6* (P < 0.01), while simultaneously upregulating anti-inflammatory factors such as *TGF-β1* and *IL-10* (P < 0.05). In contrast, the anti-inflammatory effect of EMD or GelMA alone was inferior to their composite form, suggesting a significant synergistic effect in microenvironment regulation (Zhang K. et al., 2022).

Building upon effective inflammation control, EMD-Gel further enhanced the odontogenic induction capability of DPSCs. RT-PCR and Western blot (WB) results further confirmed that the expression levels of odontogenic differentiation-related proteins such as DSPP, BSP, and RUNX2 were significantly higher in the EMD-Gel group compared to the EMD-only group and the BP control group (P < 0.01). After 21 days of mineralization induction, both ALP and Alizarin Red S (ARS) staining showed a significant increase in the area of mineralized nodules, which were 3.2 times and 5.9 times that of the control group, respectively (P < 0.01). This further validated its outstanding performance in promoting odontogenic differentiation and mineralization.

The *in vivo* experimental results similarly supported this conclusion and further confirmed the restorative advantages of EMD-Gel *in vivo*. The rat molar pulp-capping model (Yang et al., 2024) showed that in the EMD-Gel group, the inflammatory reaction was confined to the coronal pulp area at 4 days post-operation, while the radicular pulp tissue remained intact. The expression of IL-1β was significantly reduced, and DSPP was significantly upregulated (P < 0.01), indicating that the material is capable of early inflammation control and odontogenic differentiation induction (Singh et al., 2019). During the 28-day observation period, the EMD-Gel group formed a dense, continuous, and morphologically regular reparative dentin bridge (Shinde et al., 2024) with a thickness of approximately 95.3 ± 12.1 μm, which was markedly superior to the diffuse or irregular calcified layer observed in the BP group. Concurrently, inflammatory cell infiltration was significantly reduced (P < 0.05). This advantage is attributed to the dual optimization of EMD-Gel’s structure and function: its fluidity and photo-crosslinking properties ensured *in situ* shaping and sealing effectiveness (Tan et al., 2022), while the hydrogel microporous scaffold provided ideal biological support for cell migration, nutrient transport, and signal diffusion (Ma et al., 2022), thereby optimizing the outcome of the pulp capping repair.

At the molecular level, RNA-seq and PCR results showed that EMD-Gel regulates DPSC differentiation through coordinated modulation of the Wnt/β-catenin pathway and TNF-α–related inflammatory signaling. The mechanism is demonstrated as follows: the significant downregulation of *SFRP1* relieves the inhibition on the Wnt signal, promoting the nuclear translocation of β-catenin and activating the transcription of downstream odontogenic differentiation genes (Zhao et al., 2023). Simultaneously, the upregulation of *SOST* and *APCDD1* forms a negative feedback loop to prevent signal overactivation (Filipovich et al., 2011), thereby maintaining the homeostasis of the Wnt pathway and the orderly progression of the mineralization process. Furthermore, EMD-Gel optimized the microenvironment for stem cell differentiation by suppressing inflammatory signaling pathways (Zhang X. et al., 2022). It significantly reduced the expression of the inflammatory chemokine *CCL2* and the matrix metalloproteinase *MMP3*. The downregulation of *CCL2* lessens the recruitment of immune cells and the spread of the inflammatory cascade (Li et al., 2022), while the inhibition of *MMP3* mitigates ECM degradation, stabilizing the scaffold structure required for cell adhesion and differentiation (Jabłońska-Trypuć et al., 2016). Thus, EMD-Gel provides an ideal matrix environment for the sustained activation of the Wnt signal while maintaining a state of low inflammation and low degradation.

Notably, anti-inflammatory modulation and mineralization differentiation showed a strong synergistic effect within the EMD-Gel system. Excessive inflammation typically inhibits Wnt signaling (Xu et al., 2025), compromises ECM integrity through *TNF-α and IL-1β*, thereby blocking stem-cell odontogenic differentiation. EMD-Gel effectively alleviated this inhibitory step by downregulating *CCL2* and *MMP3*, allowing the Wnt signal to remain continuously active and drive DPSCs to differentiate toward the odontoblast lineage (Kornsuthisopon et al., 2024). Subsequently, the expression of Wnt downstream effector genes (such as *DSPP, DMP1*) promotes matrix mineralization and tissue structure stabilization (Hu et al., 2024), further attenuating inflammatory stimuli, thus achieving a positive feedback loop between anti-inflammation and differentiation. Accordingly, the repair mechanism of EMD-Gel can be summarized as follows: it promotes mineralization differentiation by downregulating *SFRP1* to activate the Wnt/β-catenin pathway, while simultaneously suppressing *CCL2* and *MMP3* to reduce inflammation and matrix degradation. These two pathways form a synergistic regulation at the signaling and microenvironmental levels, collectively constructing a stable regenerative ecosystem favorable for dental pulp regeneration (Brizuela et al., 2022).

Compared with previous studies, this research demonstrates significant expansion and advancement in terms of material design, functional performance, and molecular mechanism elucidation. Early studies on EMD primarily focused on its pro-differentiation effect in periodontal tissues or hDPSCs. For instance, (Zhang et al., 2022a) reported that EMD could promote odontogenic differentiation of DPSCs by activating the MAPK signaling pathways. However, these studies mostly used liquid EMD formulations, which have limited sustained action. In contrast, this study introduced GelMA photo-crosslinkable hydrogel as a three-dimensional carrier (Choi et al., 2019; Zeng et al., 2025), achieving sustained release of EMD, effectively extending its active time window, and maintaining a stable microenvironment *in vivo*. This strategy is relatively rare in previous EMD applications.

Furthermore, compared with other studies on composite delivery systems, EMD-Gel exhibits unique advantages in balancing anti-inflammatory and odontogenic differentiation effects. For example, the EMD/β-TCP composite material constructed by Shirakata et al. (2013), while enhancing mineralization capacity, suffered from high rigidity and lacked good sealing properties. This aligns with previous findings that scaffolds containing substantial inorganic components can enhance mineralization and stiffness but may reduce flexibility and interfacial sealing performance (Nooeaid et al., 2012). In comparison, the EMD-Gel in this study possesses good plasticity and moderate mechanical strength, and establishes a balance between pro-differentiation and anti-inflammation. Although several recent bioactive hydrogel systems have begun to combine immunomodulatory or anti-inflammatory properties with pro-mineralization cues, these platforms frequently rely on inorganic or nanoparticle-based components and do not consistently outperform conventional capping materials with respect to reparative dentin bridge quality (Zhang X. et al., 2024; Zhang Y. et al., 2024; Islam et al., 2025). By contrast, the EMD-Gel system enables sustained delivery of EMD within a photocrosslinkable and degradable GelMA matrix, achieving coordinated suppression of inflammation and robust odontoblast differentiation under inflammatory conditions, and, importantly, generating a continuous and structurally superior reparative dentin bridge compared with iRoot BP Plus.

At the level of signal mechanisms, the findings of this study also expand the understanding of EMD’s mode of action. Existing research often focuses on a single pathway, such as Mrunal et al. (2024), Nokhbe et al. (2012) proposing that EMD alleviates inflammatory response by inhibiting the NF-κB pathway. However, through systematic RNA-seq analysis, this study found that EMD-Gel can synergistically regulate the Wnt/β-catenin and TNF-α signaling axes, forming a bidirectional feedback loop (Nokhbe et al., 2012). This type of signaling interaction regulation mechanism has been rarely reported, suggesting that EMD-Gel possesses unique biological advantages in the field of pulp repair and regeneration.

## 4 Materials and methods

### 4.1 EMD drug concentration screening

#### 4.1.1 Cell counting Kit-8 (CCK-8) assay

The effects of EMD on DPSC proliferation were assessed using a CCK-8 kit (Dojindo, Japan). DPSCs were seeded into wells and cultured in complete medium containing EMD (0, 10, 25, or 50 μg/mL) for 24–72 h. The optical density (OD) at 450 nm was measured according to the kit instructions, and growth curves were plotted.

#### 4.1.2 RT-PCR analysis of EMD’s effect on DPSC mineralization

hDPSCs were seeded and cultured in osteogenic medium for 14 days, supplemented with different concentrations of EMD (10, 25, or 50 μg/mL). The osteogenic medium consisted of complete medium or α-MEM medium supplemented with 2% fetal bovine serum (FBS), 10 mM β-glycerophosphate, 50 μg/mL ascorbic acid, and 10 nM dexamethasone. Total RNA was extracted using a Total RNA Extraction Kit (GOONIE, Shanghai, China) following the manufacturer’s protocol. cDNA was synthesized by reverse transcription using PrimeScript RT Master Mix (TaKaRa, Japan). The mRNA expression levels of odontogenic differentiation marker genes (*OCN, DSPP, RUNX2*) were detected using the SYBR Green method (Yeasen), with GAPDH as the internal reference gene. Relative expression levels were calculated using the 2^−ΔΔCt method. Primer sequences are detailed in Supplementary Table S1.

### 4.2 Construction and characterization of EMD-Gel

#### 4.2.1 Cell counting Kit-8 (CCK-8) assay

The effects of EMD-Gel on DPSC proliferation were assessed using a CCK-8 kit (Dojindo, Japan). DPSCs were seeded into wells and cultured in complete medium containing EMD-Gel. The EMD concentration in all groups was unified at 10 μg/mL, while the GelMA concentration was varied (5%, 7.5%, or 10%). Cells were cultured for incubation periods of 24–72 h. The optical density (OD) at 450 nm was measured according to the kit instructions, and growth curves were plotted.

#### 4.2.2 Preparation and storage of sterile GelMA solution

LAP photoinitiator was first dissolved in PBS under light-protected conditions. GelMA powder was then added in different amounts to the LAP-containing PBS to obtain 5%, 7.5%, and 10% (w/v) solutions. The mixtures were sterilized through a 0.22 µm filter (Millipore, United States), aliquoted, and stored at −20 °C.

#### 4.2.3 Fabrication of EMD-Gel material

EMD (*Straumann® Emdogain®*, Basel, Switzerland) with an initial concentration of 30 mg/mL was lyophilized and ground into powder. The EMD powder was then dissolved and homogenized in the GelMA solution to form EMD-Gel. The mixture was solidified by uniform irradiation for 30 s using a portable violet light source (405 nm, 25 mW cm^-2^).

#### 4.2.4 SEM microscopic characterization

EMD-Gel of different concentrations was injected into 1 mL transparent cylindrical molds and photocured. The cured hydrogels were then frozen in liquid nitrogen and lyophilized using a freeze dryer (Martin Christ GmbH, Germany). After sputter-coating with gold, the pore structure was observed and imaged using a scanning electron microscope (FEI QUANTA 200, United States).

#### 4.2.5 Stiffness testing

This test was performed to determine the compressive modulus of EMD-Gel. Samples were prepared as EMD-Gel hydrogels measuring 2 mm × 10 mm×10 mm. After ensuring a flat surface, the samples were tested using a universal testing machine. Before testing, high-vacuum silicone grease was uniformly applied to the upper and lower compression plates to eliminate boundary effects, and the sample was placed centrally. The parameters were set as follows: compression rate of 1 mm/min and a trigger force of 0.01 N. The equipment was started, and the load-displacement data was collected in real-time at a sampling frequency of 10 Hz. After data acquisition, the maximum com-pressive strength of the hydrogel was averaged from the results of three samples. All measurements were repeated on three samples.

#### 4.2.6 Rheological testing

Rheological testing was performed on two groups: 5% EMD-Gel and Gel. The experiments were conducted using a DHR-1 rheometer equipped with a 20 mm diameter parallel plate geometry. The gap between the plates was maintained at 500 µm during testing, and measures were taken to prevent sample dehydration. All gelation kinetics experiments were performed at 37 °C with a fixed frequency of 1 Hz and a constant strain of 0.1%. This constant strain value was confirmed to be within the material’s linear viscoelastic region (LVER) during preliminary strain sweep experiments, ensuring that the measurement results were structure independent (Young et al., 2020). Furthermore, after gel curing was complete, a frequency sweep was performed at the same strain and frequency. By recording the change in G′ and G″ with frequency, the viscoelasticity of the hydrogel and the stability of its gel structure were evaluated. Photopolymerization started at 40 s.

#### 4.2.7 Degradation rate analysis

- PBS Degradation: EMD-Gel samples were prepared and lyophilized as described above, and their initial dry weight was recorded as W_0_. An identical volume of PBS was added to each well containing a hydrogel, and the samples were incubated at 37 °C. Hydrogels were retrieved at 5, 10, 15, 20, 25, and 30 days, dried, and weighed (W_1_). The degradation rate was calculated as follows:

$$\text{Degradation Rate }\left(\%\right)=\left[\left(W_{0}\;‐\;W_{1}\right)\;/\;W_{0}\right]\times 100\%$$

- Degradation in Collagenase Type II: EMD-Gel samples were prepared as above, fully swollen in PBS, dried, and weighed (W_2_). Each well was supplemented with Collagenase Type II (1U/mL, Biofroxx, Germany) and incubated at 37 °C. Hydrogels were retrieved at 2, 4, 6, 8, and 10 h, dried, and weighed (W_3_). The degradation rate was calculated as follows:

$$\text{Degradation Rate }\left(\%\right)=\left[\left(W_{2}\;‐\;W_{3}\right)\;/\;W_{2}\right]\times 100\%$$

#### 4.2.8 Swelling ratio measurement

EMD-Gel samples were prepared using the method above, weighed (recorded as W_a_), and then transferred to 6-well plates. An equal volume of PBS was added, and the hydrogels were incubated at 37 °C. Hydrogels were retrieved at 6, 12, 18, 24, 30, 36, 42, and 48 h, dried, and weighed (W_β_). The swelling ratio was calculated as follows:

$$\text{Swelling Ratio }\left(\%\right)=\left[\left(W_{\beta }\;‐\;W_{a}\right)\;/\;W_{a}\right]\times 100\%$$

#### 4.2.9 Drug release profile measurement

Amelogenin release from EMD-Gel was quantified using a commercial ELISA kit (Amelogenin ELISA Kit, SAB, China). The experiment was strictly conducted according to the manufacturer’s instructions. The concentration of amelogenin released at each time point (1, 3, 7, 12, 18, and 24 days) was determined by quantitative analysis and comparison with a standard curve.

### 4.3 Effect of EMD-Gel on inflammation and odontogenic differentiation of DPSCs

#### 4.3.1 Isolation, culture, and passage of human dental pulp stem cells (hDPSCs)

The collection and use of human dental pulp samples were approved by the Medical Ethics Committee of the Hospital of Stomatology, Sun Yat-sen University (Approval No. KQEC-2025-001-01). All donors provided written informed consent prior to sample collection. As described in our previous literature (Agha‐Hosseini et al., 2010), hDPSCs were isolated from 18- to 25-year-old healthy volunteers (n = 6) with intact third molars extracted for orthodontic reasons. Cells were cultured in α-MEM medium (GIBCO, Australia) supplemented with 20% fetal bovine serum (FBS; Gibco) and 1% penicillin-streptomycin (P/S; Sigma, United States) at 37 °C in a 5% CO_2_ high-humidity environment. The medium was replaced every 3 days and hDPSCs at passages 3–5 were used for following experiments. To evaluate the osteogenic differentiation potential of stem cells, hDPSCs underwent induction for 21 days with osteogenic medium (Cyagen, China) and stained with Alkaline Phosphatase (ALP) Staining (Sigma-Aldrich, United States) and alizarin red S (ARS) solution (Cyagen).

All methods involving human samples were performed in accordance with the relevant guidelines and regulations, and are reported in accordance with the Declaration of Helsinki and institutional requirements.

#### 4.3.2 Flow cytometry analysis of stem cell characteristics

Referring to previously established protocols (Karamzadeh et al., 2012), hDPSCs were resuspended at 10^6^ cells/100 μL. The cell suspension was incubated with Fc blocker (BioLegend, United States) for 10 min. Appropriate antibodies were then added, and the mixture was incubated in the dark at 4 °C for 30 min. Cells were resuspended in PBS and analyzed using a flow cytometer (FACScan, United States). Data analysis was performed using FlowJo *v*10.0 software (Tree Star, United States).

#### 4.3.3 Preparation of iRoot BP plus eluate

The iRoot BP Plus eluate was prepared according to methods reported in the literature (Zeng et al., 2023). The finished iRoot BP Plus product was spread flat in a culture dish to form a thin circular disc and then placed in an incubator at 37 °C, 5% CO_2_, and 100% humidity for complete curing for 2 days. After curing, it was ground into a powder, 1.000 g was weighed, and then immersed in 50 mL of αMEM in an incubator for 1 day. The solution was centrifuged at 3,000 *g* for 5 min, and the supernatant was collected. The resulting eluate was filtered through a 0.22 µm filter for sterilization and stored at 4 °C in a refrigerator. This sterile iRoot BP Plus eluate was subsequently used as the positive control group, named the BP group.

#### 4.3.4 Construction of an inflammatory model and evaluation of EMD-Gel anti-inflammatory capacity

hDPSCs seeded in plates were stimulated with 1 μg/mL *E. coli* LPS (Sigma-Aldrich, United States) for 3 h to construct an inflammatory cell model, based on the study by Xie et al. (2024). LPS-stimulated hDPSCs without hydrogel served as the negative control. mRNA expression levels of inflammation-related genes (*IL-1β, IL-6, TNF-α, IL-10*) were detected using the RT-PCR method described above (Refer to previous section number, e.g., Section 1.7), with *GAPDH* as the internal reference gene. Relative expression was calculated using the 2^ΔΔCt method. Primer sequences are detailed in Supplementary Table S1. Experimental groups included: experimental group (EMD-Gel), control groups (EMD group, GelMA group), negative control group (LPS group), positive control group (BP group) and blank control group.

#### 4.3.5 RT-PCR analysis of EMD-Gel effect on odontogenic-related gene expression

Under the aforementioned constructed inflammatory model state, hDPSCs seeded on EMD-Gel were cultured in osteogenic medium for 14 days. mRNA expression levels of odontogenic differentiation marker genes (*RUNX2, DSPP, DMP-1, ALP, BSP, OCN*) were then assessed by RT-PCR. The experimental group is consistent with the above de-scription. Primer sequences are detailed in Supplementary Table S1.

#### 4.3.6 Western blot analysis of odontogenic-related protein expression

Proteins were extracted from hDPSCs at day 14 using RIPA lysis buffer (CWBio, China) containing 1% PMSF (CWBio). Protein concentration was determined using a BCA Protein Assay Kit (CWBiotech, Beijing, China). Equal amounts of total protein were separated by electrophoresis on 4%–20% SDS-PAGE gels (ACE Biotechnology, Shanghai, China) and transferred to PVDF membranes (Millipore, United States). Membranes were blocked with 5% BSA (Sigma-Aldrich, United States) for 1 h, followed by incubation overnight at 4 °C with primary antibodies against β-Actin (Bioss Antibodies, 1:1000) and odontogenic differentiation markers: DSPP (ABclonal, 1:500), RUNX2 (ABclonal, 1:500), and BSP (ABclonal, 1:1000). After washing with Tris-buffered saline containing Tween-20 (TBST, Beyotime, Shanghai, China), membranes were incubated with horseradish peroxidase (HRP)-conjugated goat anti-rabbit/mouse secondary antibodies (Bioss Antibodies, 1:1000) at room temperature for 1 h. Signals were observed using a Western blotting detection system (Bio-Rad, United States). Analysis was performed using ImageJ (V1.8.0; NIH Image Software, United States). The experimental group is consistent with the above description.

#### 4.3.7 Effect of EMD-Gel on mineralization of DPSCs

DPSCs were seeded in plates either containing EMD-Gel (EMD-Gel group) or without EMD-Gel (CON group). After 21 days of induction in the aforementioned osteogenic differentiation medium, Alizarin Red S (ARS) and Alkaline Phosphatase (ALP) staining were performed, respectively.

- Alizarin Red S (ARS) Staining: Cells were fixed with 4% paraformaldehyde. ARS staining solution (Solarbio, Beijing, China) was added and incubated at 37 °C for 30 min protected from light. After washing with ultrapure water, red calcium deposition nodules were observed.
- Alkaline Phosphatase (ALP) Staining: Using a BCIP/NBT Alkaline Phosphatase Color Development Kit (Sigma-Aldrich, United States) according to the manufacturer’s instructions, fixed cells were incubated with the substrate solution protected from light for 15–30 min until blue precipitate developed.

### 4.4 Animal model experiments

All animal experiments were approved by the Animal Ethics Committee of Sun Yat-sen University (Approval No.: SYSU-IACUC-2024-003117). Twenty healthy 8-week-old male Sprague–Dawley rats (weighing 200–300 g) were purchased from the Experimental Animal Center of Sun Yat-sen University (Guangzhou, China) and randomly divided into four groups: Blank, LPS, iRoot BP Plus (Innovative BioCeramix, Canada), and EMD-Gel. All surgical procedures were performed under general anesthesia induced by intraperitoneal injection of Shutai® anesthetic (0.1–0.12 mL/100 g body weight). Figure 4A illustrates the procedure for establishing the rat model of experimental reversible pulpitis. The Blank group received no additional treatment. After anesthesia, pulp exposure was performed on the occlusal surface of the mandibular first molars using a high-speed turbine round bur (MANI, Japan) under continuous irrigation with sterile distilled water to prevent thermal damage to the pulp. To establish a rat model of reversible pulpitis, 2 μL of LPS solution (10 mg/mL, Sigma-Aldrich, United States) was injected into the dental pulp (Li et al., 2021). Subsequently, the exposed pulp chamber was rinsed with saline to clear hemorrhage, and a sterile cotton pellet was applied gently to control bleeding. For the LPS group, the cavity was directly covered with resin (Shofu, Japan) after hemorrhage control. For the treatment groups, the exposed pulp was directly covered with the respective iRoot BP Plus or EMD-Gel material. All cavities were then sealed with flowable resin. At the end of the experimental period, rats were euthanized under deep anesthesia by cervical dislocation, and the jaw bone samples were collected.

All animal procedures were performed in accordance with the guidelines and regulations of the Institutional Animal Care and Use Committee (IACUC) of Sun Yat-sen University. The experimental protocol was reviewed and approved by the IACUC of Sun Yat-sen University (Approval No. SYSU-IACUC-2024–003117). All methods are reported in accordance with the ARRIVE guidelines (https://arriveguidelines.org).

### 4.5 Histological analysis

Tissue samples were fixed in 4% paraformaldehyde (Sigma, United States) for 24 h. All operated teeth were scanned using a high-resolution micro-CT system (SCANCO μCT50, SCANCO Medical AG, Brüttisellen, Switzerland) with the following parameters: 10 μm voxel size, 70 kV, 200 μA, 1,800 s scan time, and 1,500 ms/frame exposure time. Analysis was performed using 3-matic Medical software v13.0 (n = 6 slices/group). Subsequently, samples were decalcified in 10% EDTA solution (Solarbio, China) under agitation for 28 days. After dehydration through a graded ethanol series, they were embedded in paraffin, and 4 μm continuous sections were prepared. H&E staining was performed strictly according to the kit instructions (Beyotime, China). Stained sections were observed under a microscope (n = 6 slices/group).

For immunohistochemistry (IHC), sections underwent microwave-mediated antigen retrieval in sodium citrate buffer (pH 6.0, Beyotime, China). After blocking with 5% BSA (Sigma, United States) for 30 min, sections were incubated overnight at 4 °C with primary anti-bodies: rabbit polyclonal anti-DSPP antibody (ABclonal, 1:200) and rabbit monoclonal anti-IL-1β antibody (Abcam, 1:500). Sections were then incubated with HRP-conjugated anti-rabbit IgG secondary antibody (Bioss, 1:1000) at 37 °C for 1 h. Signal development was performed using a DAB chromogen kit (Beyotime, China), with reaction time carefully controlled. Sections were counterstained with hematoxylin and mounted. Positive signals were quantified using ImageJ software (n = 6 slices/group).

### 4.6 RNA sequencing and RT-PCR analysis of EMD-Gel effect on key gene expression levels

Total RNA was extracted using TRIzol reagent according to the manufacturer’s instructions. RNA purity and concentration were assessed using a NanoDrop 2000 spectrophotometer (Thermo Scientific, United States). RNA integrity was evaluated using an Agilent 2100 Bioanalyzer (Agilent Technologies, Santa Clara, CA, United States). Transcriptome libraries were constructed using the VAHTS Universal V10 RNA-seq Library Prep Kit (Premixed Version) following the manufacturer’s protocol. Transcriptome sequencing and bioinformatic analysis were performed by OE Biotech Co., Ltd. (Shanghai, China). The raw RNA-seq data have been deposited in the Genome Sequence Archive for Human (GSA-Human) under accession number HRA014125 (BioProject: PRJCA049224). The data submission follows the recommended standards of the GSA-Human archive and NGDC reports (Bao et al., 2025; Zhang et al., 2025).

RT-PCR was used to verify the effect of EMD-Gel on the expression levels of key genes such as *SOST, APCDD1, CCL2, MIMP3,* and *SFRP1* after 14 days of mineralization induction. The experimental groups were the Control Group, consisting of mineralization induction medium only, and the EMD-Gel Group, which utilized LPS combined with mineralization induction medium and EMD-Gel. All cells were cultured in mineralization induction medium for 14 days to investigate the influence of EMD-Gel on cell mineralization and anti-inflammatory pathways. Primer sequences are detailed in Supplementary Table S1.

### 4.7 Statistical analysis

The sample size for each statistical analysis was n ≥ 3. All data are presented as mean ± standard deviation (SD). One-way analysis of variance (ANOVA) was performed using SPSS software to determine statistically significant differences between groups. A p-value <0.05 was considered statistically significant.

## 5 Conclusion

In summary, EMD-Gel achieves a synergistic effect of anti-inflammation and odontoblast differentiation induction by activating the Wnt/β-catenin pathway and inhibiting the *CCL2*-*MMP3*-mediated inflammatory response, thus creating a functional microenvironment conducive to the homeostatic regeneration of pulp tissue (Gong et al., 2016). *In vivo*, EMD-Gel demonstrated clear advantages relative to iRoot BP Plus, forming a more continuous and better-organized reparative dentin bridge and achieving more effective suppression of inflammatory infiltration, thereby indicating comparatively enhanced regenerative performance. This composite scaffold demonstrates significant advantages in physical properties, biological activity, and signal regulation, providing new theoretical basis and material foundation for vital pulp treatment and pulp regeneration. We will next investigate the temporal sequence and overall synergy among the signaling pathways. Concurrently, a systematic evaluation of its long-term application potential, spanning biosafety, functional integration, and clinical feasibility, must be performed to solidify the foundation for its clinical use in dental pulp bioregeneration.

## Data Availability

The raw sequencing data reported in this study have been deposited in the Genome Sequence Archive for Human (GSA-Human) in the National Genomics Data Center, China National Center for Bioinformation/Beijing Institute of Genomics, Chinese Academy of Sciences under the accession number HRA014125 (BioProject accession: PRJCA049224). The dataset is publicly accessible at https://ngdc.cncb.ac.cn/gsa-human/browse/HRA014125.
