# Supplementary material for: Application and mechanism study of EMD-Gel composite scaffold in dental pulp tissue repair
Source: Front Bioeng Biotechnol. 2025 Dec 10;13:1739495. doi: 10.3389/fbioe.2025.1739495 (PMC12728041; doi:10.3389/fbioe.2025.1739495)
Supplement: Supplementary file 1 [file DataSheet1.pdf]

Abbreviations

The following abbreviations are used in this manuscript:

EMD	Enamel Matrix Derivative
GelMA	Gelatin Methacryloyl
DPSCs	Dental Pulp Stem Cells
TNF	Tumor Necrosis Factor
RNA-seq	RNA sequencing
CCL2	Chemokine (C-C motif) ligand 2
MMP3	Matrix Metalloproteinase 3
SFRP1	Secreted Frizzled-Related Protein 1
SOST	Sclerostin
APCDD1	Adenomatosis Polyposis Coli Down-Regulated 1
ALP	Alkaline Phosphatase
DSPP	Dentin Sialophosphoprotein
RUNX2	Runt-Related Transcription Factor 2
OCN	Osteocalcin
ARS	Alizarin Red Staining
WB	Western Blot
IHC	Immunohistochemistry
PBS	Phosphate-Buffered Saline

Appendix A

S.1 Stem cell characterization and material biosafety validation

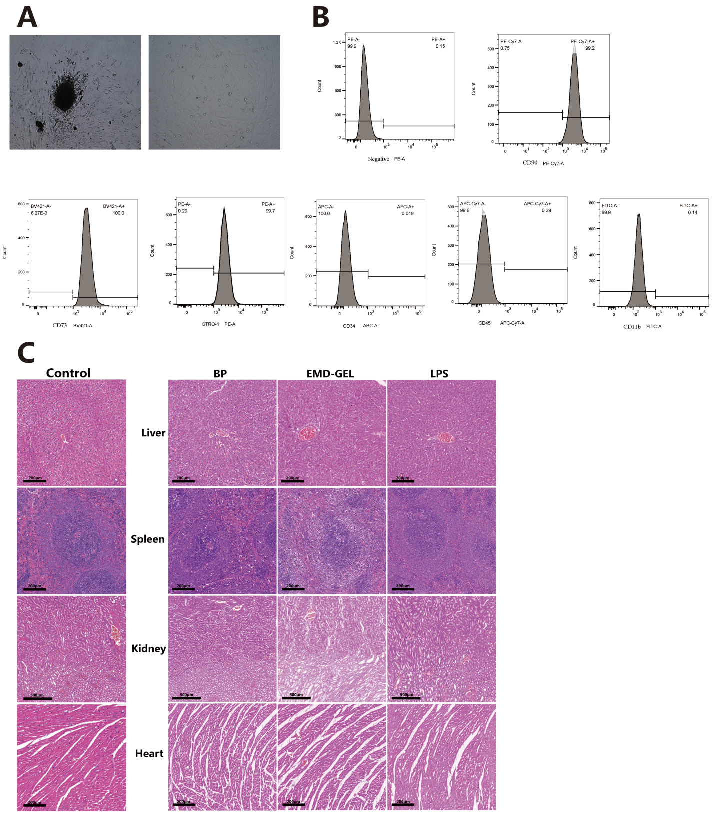

(a) Primary culture morphology of dental pulp stem cells； (b) Flow cytometry to detect mesenchymal molecular markers on the surface of hDPSCs； (c) Histopathological analysis of major organs of rats by EMD-Gel.

Table S1. Experimental RT-PCR primer and probe base sequence design.

Gene	Forward primer (5′-3′)	Reverse primer (5′-3′)
GAPDH	GCAGGAGGCATTGCTGAT	CACCATCTTCCAGGAGCGAG
RUNX2	CCGCCTCAGTGATTTAGGGC	GGGTCTGTAATCTGACTCTGTCC
DMP-1	CACTCAAGATTCAGGTGGCAG	TCTGAGATGCGAGACTTCCTAAA
DSPP	TGGCGATGCAGGTCACAAT	CCATTCCCACTAGGACTCCCA
ALP	CCCGCTTTAACCAGTGCAAC	GAGCTGCGTAGCGATGTCC
BSP	GAACCTCGTGGGGACAATTAC	CATCATAGCCATCGTAGCCTTG
OCN	CACTCCTCGCCCTATTGGC	CCCTCCTGCTTGGACACAAAG
IL-1β	AGCTACGAATCTCCGACCAC	CGTTATCCCATGTGTCGAAGAA
IL-6	ACTCACCTCTTCAGAACGAATTG	CCATCTTTGGAAGGTTCAGGTTG
TNF-α	GAGGCCAAGCCCTGGTATG	CGGGCCGATTGATCTCAGC
IL-10	TAGAGCTGCGGACTGCCTTCA	ATGCTCCTTGATTTCTGGGCCAT
TGF-β1	CTAATGGTGGAAACCCACAACG	TATCGCCAGGAATTGTTGCTG
SOST	ACACAGCCTTCCGTGTAGTG	GGTTCATGGTCTTGTTGTTCTCC
APCDD1	AGGCTGTGAAGTAAGGTCAGG	GGCCTTGAAGGTGTTATTGTGG
CCL2	CAGCCAGATGCAATCAATGCC	TGGAATCCTGAACCCACTTCT
MMP3	AGTCTTCCAATCCTACTGTTGCT	TCCCCGTCACCTCCAATCC
SFRP1	ACGTGGGCTACAAGAAGATGG	CAGCGACACGGGTAGATGG
